# The influence of penguin activity on soil diatom assemblages on King George Island, Antarctica with the description of a new *Luticola* species

**DOI:** 10.7717/peerj.13624

**Published:** 2022-07-04

**Authors:** Natalia Kochman-Kędziora, Teresa Noga, Maria Olech, Bart Van de Vijver

**Affiliations:** 1 Department of Ecology and Environmental Protection, University of Rzeszów, Rzeszów, Poland; 2 Department of Soil Studies, Environmental Chemistry and Hydrology, University of Rzeszów, Rzeszów, Poland; 3 Department of Polar Research and Documentation, Jagiellonian University Cracow, Kraków, Poland; 4 Institute of Biochemistry and Biophysics, Polish Academy of Sciences, Warszawa, Poland; 5 Research Department, Meise Botanic Garden, Meise, Belgium; 6 Department of Biology, ECOSPHERE, University of Antwerp, Antwerp, Belgium

**Keywords:** Antarctic Region, South Shetlands, King George Island, Bacillariophyta, Soil diatoms, Diversity, *Luticola kaweckae*, New species

## Abstract

**Background:**

Ice-free areas in the Antarctic region are strongly limited. The presence of marine mammals and birds in those areas influence soil properties and vegetation composition. Studies on the terrestrial diatom flora in soils influenced by sea birds in the Maritime Antarctic region are scarce.

**Methods:**

Samples were collected from two transects on the western shore of the Admiralty Bay region. Light and scanning electron microscopic observations and statistical analyses were conducted to consider the impact of penguin rookeries on soil diatom assemblages.

**Results:**

The disturbance associated with the presence of penguin rookeries clearly influences the soil diatom diversity. Assemblages from areas with the highest nutrient input were characterized by a much lower diversity with only few species dominating the flora. One of recorded taxa could not be assigned to any of the known species. Therefore, based on the combination of morphological features analyzed using light and scanning electron microscopes and comparison with similar taxa in the Antarctic region and worldwide, the species is described hereby as new to science–*Luticola kaweckae* sp.nov. The new species is characteristic for soil habitats with strong penguin influence.

## Introduction

Ice-free areas are limited to only a few percent of the entire Antarctic region, located primarily near the coastline (Spaulding et al., 2010). These terrestrial ecosystems are, in general, poor in nutrients. However, the presence of marine mammals and birds, such as penguins and elephant seals, can highly fertilize soil, resulting in an increased nitrogen and salinity input associated with animal activity, sea spray and nitrogen-fixing autotrophs. Breeding penguins are the dominant contributor to fertilization in Antarctic terrestrial ecosystems (Tatur, 2002). The intensity of the fertilization during the breeding season exceeds almost 10 kg of dry excrements per square meter (Tatur & Myrcha, 1984). The amount of nutrients supplied with bird excrement is much larger than from other sources (sea aerosols, wind deposition, precipitation) (Bokhorst et al., 2007; Zwolicki et al., 2015). The huge guano deposition in penguin rookeries leads to increased phosphatization, influencing soil properties and vegetation composition (Zwolicki et al., 2015) and, as a consequence, to the development of unique, ornithogenic soils (Tatur & Myrcha, 1984; Tatur, 1992; Tatur, 2002; Simas et al., 2008). The importance of nutrients supplied by breeding birds and mammals to the Antarctic terrestrial ecosystems has been discussed in the past (*i.e*. Tatur & Myrcha, 1984; Myrcha & Tatur, 1991; Smykla et al., 2006, Smykla, Wołek & Barcikowski, 2007; Qin et al., 2014). Several studies have reported a specific vegetation zonation surrounding penguin rookeries and nesting places (Smith, 1984; Zarzycki, 1993; Olech, 2002; Smykla et al., 2006; Smykla, Wołek & Barcikowski, 2007; Zwolicki et al., 2015). Intensive trampling in an active colony together with a fertilization level largely exceeding the plant tolerances, strongly limit vegetation development. The soil is devoid of vegetation and its top layer contains, among others, a high amount of eggshells, feathers and bones. However, the surrounding area can be colonized by algae, lichens and plants. The differences in the structure of the Antarctic vegetation is related to the intensity of penguin rookery impact (Smith, 1984; Zarzycki, 1992; Olech, 2002; Smykla et al., 2006; Smykla, Wołek & Barcikowski, 2007; Zwolicki et al., 2015). Therefore, the specific vegetation zonation surrounding breeding colonies is largely related to an increasing distance from the penguin rookeries, with more complex and diverse vegetation further away from the rookeries. Moreover changes in soil properties are likewise observed. With increasing distance from the edges of the rookery, nutrients concentrations and soil conductivity decrease, while the pH increases (Zwolicki et al., 2015).

Diatoms (Bacillariophyta) are one of the most abundant and productive algal groups in Antarctic and sub-Antarctic inland waters and terrestrial environments (Jones, 1996; Van de Vijver & Beyens, 1999; Sabbe et al., 2003; Verleyen et al., 2021). Numerous studies reveal that Antarctic and sub-Antarctic diatoms are part of the unique flora exposed to the adverse climatic conditions (Verleyen et al., 2021, and references therein). Despite this, they can find favorable environmental conditions for surviving in various habitats. Freshwater diatoms form diverse communities in lakes, ponds, small water reservoirs on postglacial moraines, streams, often ephemeral, fed by melting snow and ice cover. Moreover, they also occur on moist moss vegetations and the surface soil layer, where they can often dominate the algal flora (Zidarova, Kopalová & Van de Vijver, 2016).

In the past, the lack of adequate literature for diatom identification in the Antarctic regions resulted in force-fitting (Tyler, 1996). Recent taxonomic revisions of the Antarctic diatom flora, based on a narrower species concept (see Mann, 1999), showed that the actual diatom diversity in the Antarctic Region was underestimated (Zidarova, Kopalová & Van de Vijver, 2016; Verleyen et al., 2021, and references therein).

The application of this more fine-grained taxonomy also resulted in a fresh view on Antarctic diatom biogeography, pointing to an Antarctic diatom flora that is more unique than previously accepted (Vyverman et al., 2007, 2010; Kociolek et al., 2017; Verleyen et al., 2021), composed of a large number of endemic species and subdivided in three main regions of the Antarctic realm: Continental Antarctica, Maritime Antarctic Region and the sub-Antarctic islands (Verleyen et al., 2021).

Terrestrial diatoms assemblages in soils influenced by sea birds and marine mammals are only well studied in the sub-Antarctic region (Van de Vijver & Beyens, 1998; Van de Vijver, Ledeganck & Beyens, 2002; Moravcová, Beyens & Van de Vijver, 2010). Van de Vijver, Ledeganck & Beyens (2002) primary investigated diatom assemblages in different group of soils, including biogenic soils on Ile de la Possession (Crozet Archipelago, southern Indian Ocean) and identified two main environmental factors that influenced subantarctic soil diatoms: moisture and nutrients. On the same island, Moravcová, Beyens & Van de Vijver (2010) investigated diatom assemblages within two breeding colonies of the wandering albatross (*Diomedea exulans*). There is only one article discussing the algal communities (including diatoms) in the ornithogenic soils in the Maritime Antarctic region (Mataloni & Tell, 2002), where three soil sites with different degrees of influence from the penguins colonies at Cierva Point (Antarctic Peninsula region) were investigated. As these studies were performed almost 20 years ago, they do not reflect the latest trends in diatom taxonomy and might hence incorrectly assess changes in the diversity and composition. Since the past two decades a great effort was made to revise the freshwater and terrestrial Maritime Antarctic diatom flora, we now have a better understanding of the actual diatom diversity (Zidarova, Kopalová & Van de Vijver, 2016 and references therein). Based on the obtained results, several taxa are now known to be associated with increased nutrients, mainly of biotic origin, such as *Psammothidium superpapilio*Kopalová, Zidarova & Van de Vijver (2016), *Humidophila australoshetlandica* Kopalová, Zidarova & Van de Vijver (Kopalová et al., 2015), *Luticola olegsakharovii*Zidarova, Levkov & Van de Vijver (2014) and *Pinnularia austroshetlandica* (G.W.F.Carlson) A.Cleve (Zidarova, Kopalová & Van de Vijver, 2012).

However, none of the current research deals with the structure of the diatom assemblages in the ornithogenic soils of the Maritime Antarctic Region, as they are mainly focused on the taxonomy and morphology of the individual taxa in those habitats. The only recent survey of the soil diatoms on King George Island, based on the refined taxonomy, investigated the diatom flora from soils in two stream valleys close to the Polish Arctowski Station (Noga et al., 2020). Additionally, several new species were described from King George Island (Kochman-Kędziora et al., 2016, 2017, 2018, 2020; Kochman-Kędziora, Olech & Van de Vijver, 2020). Although some of them were recorded in soils strongly influenced by penguin excrements (Kochman-Kędziora, Olech & Van de Vijver, 2020; Kochman-Kędziora et al., 2020), the diversity of soil diatoms on the penguin rookeries and the general characteristic of the assemblages are still unknown.

To improve our current knowledge of the diversity and ecology of Antarctic diatoms we conducted a study on terrestrial diatoms from an area influenced by penguins, aiming to (1) determine whether and how the presence of penguin rookeries affects the diversity and development of diatom assemblages, (2) indicate possible differences in diatom composition between two penguin rookeries, (3) increase the knowledge of the ecology of Antarctic terrestrial diatom species.

During this study, a *Luticola* taxon that could not be identified using the currently available literature was observed. Based on detailed LM and SEM observations the taxon is described herein as a new species: *Luticola kaweckae* sp. nov., comparing the new species with other taxa from the *Luticola muticopsis* group. Brief notes on its ecology are likewise presented.

## Materials and Methods

### Site descriptions

**Figure 1:**
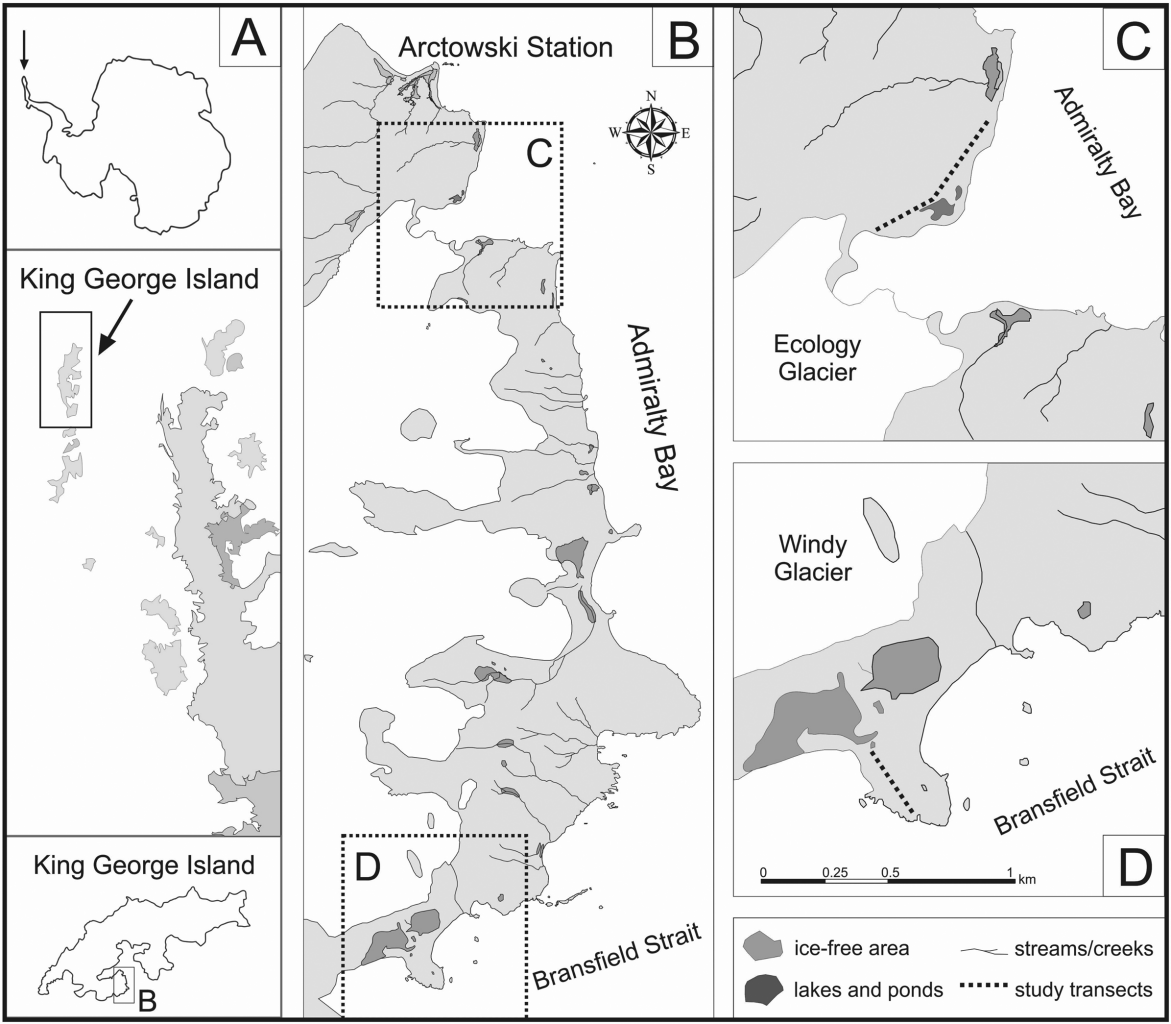
Location of study area on the western shore of the Admiralty Bay (based on Pudełko, 2003, 2008). (A) King George Island located north from the Antarctic Peninsula (B) The western shore of the Admiralty Bay with two studied transects: transect A on the Ecology Glacier Forefield (C) and transect B on the Windy Glacier Forefield (D).

The Maritime Antarctic region encompasses the west coast of the Antarctic Peninsula south to Marguerite Bay, as well as several islands and archipelagos (South Sandwich, South Orkney, South Shetland Islands, Palmer Archipelago). King George Island (61°54′–62°16′S/57°35′–59°02′W), the largest of the South Shetland Islands, is situated about 120 km north from the northernmost part of the Antarctic Peninsula. The research was conducted on the western shore of Admiralty Bay, in the territory of the Antarctic Specially Protected Area No. 128 (ASPA 128), south from the Polish Arctowski Station (Fig. 1). The Antarctic Specially Protected Area No. 128 (ASPA 128) is a big lowland ice-free area (16.8 km^2^) located on the western shore of the Admiralty Bay Region (King George Island, South Shetland Islands). ASPA 128 was established in 1979 under the name Site of Special Scientific Interest No. 8 to protect the mosaic of valuable natural habitats, covered by diverse tundra vegetation. Together with numerous breeding and nesting places of local fauna, this protected zone is a representative part of the Maritime Antarctic ecosystem (Sierakowski, Korczak-Abshire & Jadwiszczak, 2017; Dabski et al., 2020).

King George Island has a typically maritime climate, with small annual variations in air temperature and constant cloud cover. Strong influence of oceanic air masses causes high values of air humidity (Wen et al., 1994; Rakusa-Suszczewski, 2002). The mean annual air temperature is about −2.5 °C, although temperatures above 0 °C are observed each month. Mean annual precipitation varies around 600–700 mm (Peter et al., 2008; Liu et al., 2011). Most of the island is covered by a permanent ice dome with ice-free areas being only scarce. Despite the harsh environmental conditions and ice-free areas limited to less than 10% of the island area, terrestrial ecosystem are relatively well developed (Zwolicki et al., 2015). The vegetation of King George Island is mainly composed of cryptogams: mosses, liverworts, lichens, algae and cyanobacteria with only two native species of the vascular plants (*Deschampsia antarctica* Desv. and *Colobanthus quitensis* (Kunt) Bartl). The western coast of Admiralty Bay is considered to be one of the botanically richest areas in the Maritime Antarctic region (Krzewicka & Smykla, 2004; Zwolicki et al., 2015). In the ASPA 128 area, several breeding colonies of following species have been reported: Adélie penguin (*Pygoscelis adeliae* Hombron & Jacquinot), chinstrap penguin (*P. antarcticus* Forster) and gentoo penguin (*P. papua* Forster). During the 1978–1996 period, breeding populations of all penguin species in the Admiralty Bay region significantly decreased (Sierakowski, Korczak-Abshire & Jadwiszczak, 2017), the result of several factors–reduction of sea ice cover in the Antarctic Peninsula region, decline of krill population, as well as local human disturbance, especially in the vicinity of the Polish Arctowski Research Station (Sierakowski, Korczak-Abshire & Jadwiszczak, 2017 and references therein).

### Diatom sampling

Two independent transects were investigated by Prof. M. Olech during several Antarctic Expeditions (summer seasons 2005/2006, 2006/2007, 2008/2009 and 2014/2015). The permit to enter the Antarctic Specially Protected Area No. 128 in the season 2014/2015 (No. 5/2014) was granted by the Institute of Biochemistry and Biophysics of the Polish Academy of Sciences. Transects A and B were determined in two comparable locations, including a similar distance from the sea and a similar topographic profile (Fig. 1). To consider the impact of penguin rookeries on soil diatoms, both transects start at the penguin rookery and finish on the natural topographic barrier. In order to investigate the differences in diatom composition between areas with and without penguin influence, one of the transects was prolonged and several additional samples were collected.

Transect A, approximately 600 m long, was designated along the sea shore on the lateral moraine of the Ecology Glacier Forefield. It starts on the top of a low moraine in the middle of a gentoo penguin *(Pygoscelis papua)* rookery, running parallel to the sea shore and ending in the front of Ecology Lagoon. Only the first part of the transect is influenced by the penguins. These sites are located between two low ridges of the lateral moraine. To observe possible differences in the structure of diatom communities, the second part of the transect, devoid of direct penguin influence, was also analyzed.

Transect B, approximately 250 m long, was established on a small cape, called Patelnia Point. It starts at the chinstrap penguin (*Pygoscelis antarcticus*) rookery, runs to the north and ends at a natural topographic barrier, *i.e*. the front of a small water body.

Samples were collected twice from both transects: transect A: 2007 and 2015, transect B: 2006 and 2009. On each site, samples were collected from the surface soil layer. Only at the first site in transect A in 2015 (A1.15), the sample from the penguin rookery was collected from a cyanobacterial mat of *Prasiola crispa* with small plant debris, feathers and guano. A total of 49 samples were collected during a fieldwork. Sample codes include the code of the transect from which the sample was taken (A, B) and the year of sampling (2006, 2007, 2009 or 2015).

Samples for diatom analysis were prepared following the method used by Kawecka et al. (1998) and Kawecka (2012). The sampling and analyzing methods were performed as previously described in Noga et al. (2020). Part of each soil sample was digested using a mixture of concentrated sulfuric and chromic acid. Following digestion and centrifugation (5 times 5 min at 2,500 rpm), the resulting cleaned material was mounted in Pleurax (refractive index 1.75) in order to obtain permanent diatom slides. Light microscopy (LM) observations were performed using a Nikon ECLIPSE 80i and a Carl Zeiss Axio Imager A2 equipped with Differential Interference Contrast (Nomarski) optics. Diatom images were captured using the Zeiss ICC 5 camera. For scanning electron microscopy (SEM), part of the cleaned material was filtered through a 5 μm Isopore™ polycarbonate membrane filter (Merck Millipore, Burlington, MA), air–dried and attached to aluminum stubs. The stub was subsequently sputter-coated with a 20 nm layer of Au using the Turbo-Pumped Sputter Coater Quorum Q 150OT ES and studied in a Hitachi SU8010 microscope at 5 kV at the University of Rzeszów (Poland). Cleaned diatom material and microscopic slides are stored at the University of Rzeszów. In each sample, an exact total of 350 diatom valves were counted along random transects. After each 350 valve count, the remainder of the slide was scanned for rare species that were not observed during the counting. When due to the low abundance of diatom valves in the slide, it was impossible to count 350 specimens in random views, an entire slide was scanned and all observed specimens in the slide were enumerated. Diatom identification was based on Zidarova, Kopalová & Van de Vijver (2016 and references listed therein), Kochman-Kędziora, Olech & Van de Vijver (2020) and Kochman-Kędziora et al. (2017, 2020). Marine taxa were identified using Witkowski, Lange-Bertalot & Metzeltin (2000); Al-Handal & Wulff (2008); Al-Handal et al. (2008) and Al-Handal, Riaux-Gobin & Wulff (2010).

Soil pH and conductivity (EC) were measured in the laboratory using pH meter with application of suspension of 1:2.5 soil to distilled water ratio using a MARTINI PH65 meter and a MARTINI EC59 meter. When the conductivity values exceeded the limit of quantification of the handheld MARTINI EC59 meter, the measurements were performed with a laboratory conductometer HANNA HI 2316. The measurements were performed for samples collected in three seasons. Due to logistical reasons, for the samples collected in 2006 from transect B (B1.06–B9.06) pH and EC were not measured.

### Data analysis

Prior to statistical analysis, rare taxa (*i.e*. a taxon not present in at least one sample with a minimum relative abundance of 1%) were removed from all further statistical analyses, a common practice in multivariate studies dealing with large number of taxa (Kopalová et al., 2013) as most of these taxa may represent accidental occurrences, the result of uncertain taxonomy or contamination. Samples containing less than 100 valves (15 of the 49 samples) were also removed prior to further analysis, based on Bate & Newall (2002) showing that a count size of 100 valves is acceptable to detect community patterns using statistical multivariate techniques. To visualize multivariate patterns in the species composition, detrended correspondence analysis (DCA) was performed using Canoco 5 (Ter Braak & Šmilauer, 2012).

A preliminary analysis indicated the presence of one clear outlier (A5.07). The diatom community of sample A5.07 was formed by *Chamaepinnularia krookiiformis* (Krammer) Lange-Bertalot & Krammer, following by *Pinnularia microstauroides* Zidarova, Kopalová & Van de Vijver and *Mayamaea permitis* (Hustedt) Bruder & Medlin. As this assemblage was not observed elsewhere in the studied samples, sample A5.07 was removed as outlier before further DCA analysis.

The gradient length of the DCA analysis was 3.6 standard deviation (SD) justifying the use of unimodal analyses (Ter Braak & Prentice, 1988). The first two ordination axes explained 39.6% (axis 1 = 15.9%, axis 2 = 23.7%) of the cumulative variation in the diatom data set. with an additional 27.8% on the third axis.

A permutation analysis of variance (PERMANOVA) was used on the Bray-Curtis similarity matrix with 999 permutations to test weather diatom assemblages differ in the samples influenced and non-influences by penguins, as well as between samples collected from two different penguin rookeries (transect A and B). The level of significance for the test was set as *P* < 0.05. Diatom data were square-root transformed prior to PERMANOVA analysis.

The Shannon’s diversity (H’) and Pielou’s evenness (J’) were calculated for samples, where 350 specimens were counted. The permutation analysis of variance, Shannon’s diversity (H’) and Pielou’s evenness (J’) indices were performed using PRIMER v.7 software (Anderson, Gorley & Clarke, 2008).

## Results

### Diatom richness

In total, 94 diatom taxa (including species, subspecies and varieties) have been recorded in 49 samples (Table 1). However, the number of recorded species and counted valves in individual samples was not equal. In half of the samples (25), less than ten species were recorded, whereas in only five samples the number of recorded species exceeded 20. The highest species richness was observed in sample A9.07 with 29 taxa. An unknown *Luticola* species, described in the present paper as *Luticola kaweckae* sp. nov. reached 36.2% of all valves counted during the study.

**Table 1:** List of the recorded taxa with their abundances (%) in samples influenced (first part of transect A, transect B) and not-influenced by penguins (second part of transect A).

Taxon name	Transect B	Transect A: samples with penguin influence	Transect A: samples devoid of penguin influence
*Achnanthes coarctata* (Brébisson) Grunow	0	0	2.5
*Achnanthes kohleriana* Kopalová, Zidarova & Van de Vijver	0	0	0.9
*Achnanthes muelleri* G.W.F.Carlson	0.2	0	+
*Achnanthes tayloreniss* D.E.Kellogg et al.	0	0	1.4
*Chamaepinnularia gerlachei* Van de Vijver & Sterken	0.6	+	4.9
*Chamaepinnularia krookiformis* (Krammer) Lange-Bertalot & Krammer	3.8	3.9	7.7
*Fragilaria* cf. *parva* Tuji & D.M.Williams	+	0	0
*Hantzschia abundans* Lange-Bertalot	0	0	2.0
*Hantzschia amphioxys* (Ehrenberg) Grunow	+	0	3.6
*Hantzschia amphioxys* f. *muelleri* Ts.Ko-Bayashi	0	0	0.1
*Hantzschia hyperaustralis* Van de Vijver & Zidarova	0	0	0.1
*Hippodonta hungarica* (Grunow) Lange-Bertalot, Metzeltin & Witkowski	0	0	0.1
*Humidophila keiliorum* Kopalová	0	0	+
*Humidophila sceppacuerciae* Kopalová	0	0	0.8
*Humidophila vojtajarosikii* Kopalová, Zidarova & Van de Vijver	0	+	1.8
*Humidophila tabellariaeformis* (Krasske) R.L.Lowe et al.	0	+	0
*Luticola amoena* Van de Vijver, Kopalová, Zidarova & Levkov	1	0	0
*Luticola andina* Levkov, Metzeltin & Pavlov	+	0	0.5
*Luticola australomutica* Van de Vijver	0.2	0	2.4
*Luticola austroatlantica* Van de Vijver et al.	0	0.1	1.4
*Luticola* cf. *australomutica*	0.6	0	0.7
*Luticola contii* Zidarova, Levkov & Van de Vijver	0	0	0.8
*Luticola katkae* Van de Vijver & Zidarova	0	0	0.2
*Luticola kaweckae* sp. nov.	51.2	54.8	7.2
*Luticola muticopsis* (Van Heurck) D.G.Mann	1.0	0.4	0.7
*Luticola olegsakharovii* Zidarova, Levkov & Van de Vijver	0.8	1.6	1.4
*Luticola permuticopsis* Kopalová & Van de Vijver	0	0	+
*Luticola puchalskiana* Kochman-Kędziora et al.	9.1	0	0
*Luticola pusilla* Van de Vijver, Kopalová, Zidarova & Levkov	0	0	0.3
*Luticola quadriscrobiculata* Van de Vijver	0	0	8.5
*Luticola truncata* Kopalová & Van de Vijver	0	+	+
*Luticola vandevijveri* Kopalová, Zidarova & Levkov	0	0	+
*Luticola* sp. 1	0	0	0.2
*Luticola* sp. 2	0	0	0.1
*Luticola* sp. 3	0	0	0.1
*Luticola* sp. 4	0	0	0.2
*Luticola* sp. 5	+	0	0
*Mayamaea* cf. *atomus* (Hustedt) Bruder & Medlin	0	0.1	0.5
*Mayamaea permitis* (Hustedt) Bruder & Medlin	0	1.7	0
*Muelleria australoatlantica* Van de Vijver & S.A.Spaulding	0	0	0.7
*Muelleria kristinae* Van de Vijver	0	0	+
*Muelleria olechiae* Kochman-Kędziora et al.	0	0	0.5
*Navicula australoshetlandica* Van de Vijver	0	0.1	1.6
*Navicula dobrinatemniskovae* Zidarova & Van de Vijver	0	0	0.2
*Navicula gregaria* Donkin	0	0	5.3
*Navicula massalskiana* Kochman-Kędziora, Olech & Van de Vijver	2.3	0	0
*Navicula romanedwardii* Zidarova, Kopalová & Van de Vijver	0	0	0.6
*Nitzschia annewillemsiana* Hamsher et al.	0	0	+
*Nitzschia gracilis* Hantzsch	0	+	+
*Nitzschia homburgiensis* Lange-Bertalot	0	0.3	0.5
*Nitzschia kleinteichiana* Hamsher et al.	0	0	10.2
*Pinnularia australoborealis* Van de Vijver & Zidarova	4.0	1.4	0.4
*Pinnularia australoglobiceps* Zidarova, Kopalová & Van de Vijver	+	0	0
*Pinnularia australomicrostauron* Zidarova, Kopalová & Van de Vijver	1.1	1.5	0.6
*Pinnularia australoschoenfelderi* Zidarova, Kopalová & Van de Vijver	0.8	+	0.3
*Pinnularia austroshetlandica* (Carlson) Cleve-Euler	8.2	0	0
*Pinnularia borealis* Ehrenberg s.l.	11.4	25.8	22.6
*Pinnularia borealis* Ehrenberg var. *scalaris (*Ehrenberg) Rabenhorst	0	0.4	1.9
*Pinnularia borealis* Ehrenberg var. *pseudolanceolata* Van de Vijver & Zidarova	0	0	5.1
*Pinnularia magnifica* Zidarova, Kopalová & Van de Vijver	0	0	+
*Pinnularia microstauroides* Zidarova, Kopalová & Van de Vijver	0	2.7	0
*Pinnularia perlanceolata* Van de Vijver & Zidarova	0	0	0.2
*Pinnularia rabenhorstii* var. *subantarctica* Van de Vijver & Le Cohu	0	+	0
*Pinnularia strictissima* Manguin	0.3	0	0
*Pinnularia subantarctica* var. *elongata* (Manguin) Van de Vijver & Le Cohu	2.6	0.5	1.5
*Pinnunavis gebhardii* (Krasske) Van de Vijver	+	0	0
*Placoneis australis* Van de Vijver & Zidarova	+	0.2	0.2
*Planothidium australe* (Manguin) Le Cohu	0	+	0.4
*Planothidium rostrolanceolatum* Van de Vijver, Kopalová & Zidarova	0	0.1	+
*Psammothidium germainii* (Manguin) Sabbe	0.3	2.3	2.3
*Psammothidium papilio* (D.E.Kellogg et al.) Kopalová & Van de Vijver	+	0	0
*Psammothidium rostrogermainii* Van de Vijver, Kopalová & Zidarova	+	0	1.9
*Sellaphora nana* (Hustedt) Lange-Bertalot et al.	0	0	+
*Stauroneis husvikensis* Van de Vijver & Lange-Bertalot	0	0	+
*Stauroneis latistauros* Van de Vijver & Lange-Bertalot	0	0	+
*Stauroneis minutula* Hustedt	0	0	0.1
*Stauroneis pseudomuriella* Van de Vijver & Lange-Bertalot	0	0	+
marine species			
*Cocconeis costata* group W.Gregory	0.2	0.6	0.2
*Cocconeis dallmannii* Al-Handal, Riaux-Gobin & Wulff	0	0.2	0
*Cocconeis japonica* var. *antarctica* H.F.Van Heurck 1909:	0	0	+
*Cocconeis melchioroides* Al-Handal et al.	+	0.1	+
*Cocconeis pinnata* var. *matsii* Al-Handal, Riaux-Gobin & Wulff	0	+	0.1
*Fragilariopsis curta* (Van Heurck) Hustedt	0.1	+	0
*Fragilariopsis kerguelensis* (O’Meara) Hustedt	0.1	0.1	0
*Fragilariopsis *cf.* rhombica*	0	0.2	0
*Licmophora* sp.	+	0	0.1
*Navicula perminuta* Grunow	0.6	0.1	0.1
*Navicula salinarum* Grunow	0	0	0.4
*Navicula* sp.	0	+	0
*Pseudogomphonema kamtschaticum* (Grunow) Medlin	0	0.1	0
*Pseudogomphonema* sp.	+	0	0
*Thalassiosira gracillis* (Karsten) Hustedt	0.5	0.4	0.4
Sum of taxa	35	37	71

**Note:**

+ single valve of species was observed.

### Novel taxon–diagnosis

**Figure 2:**
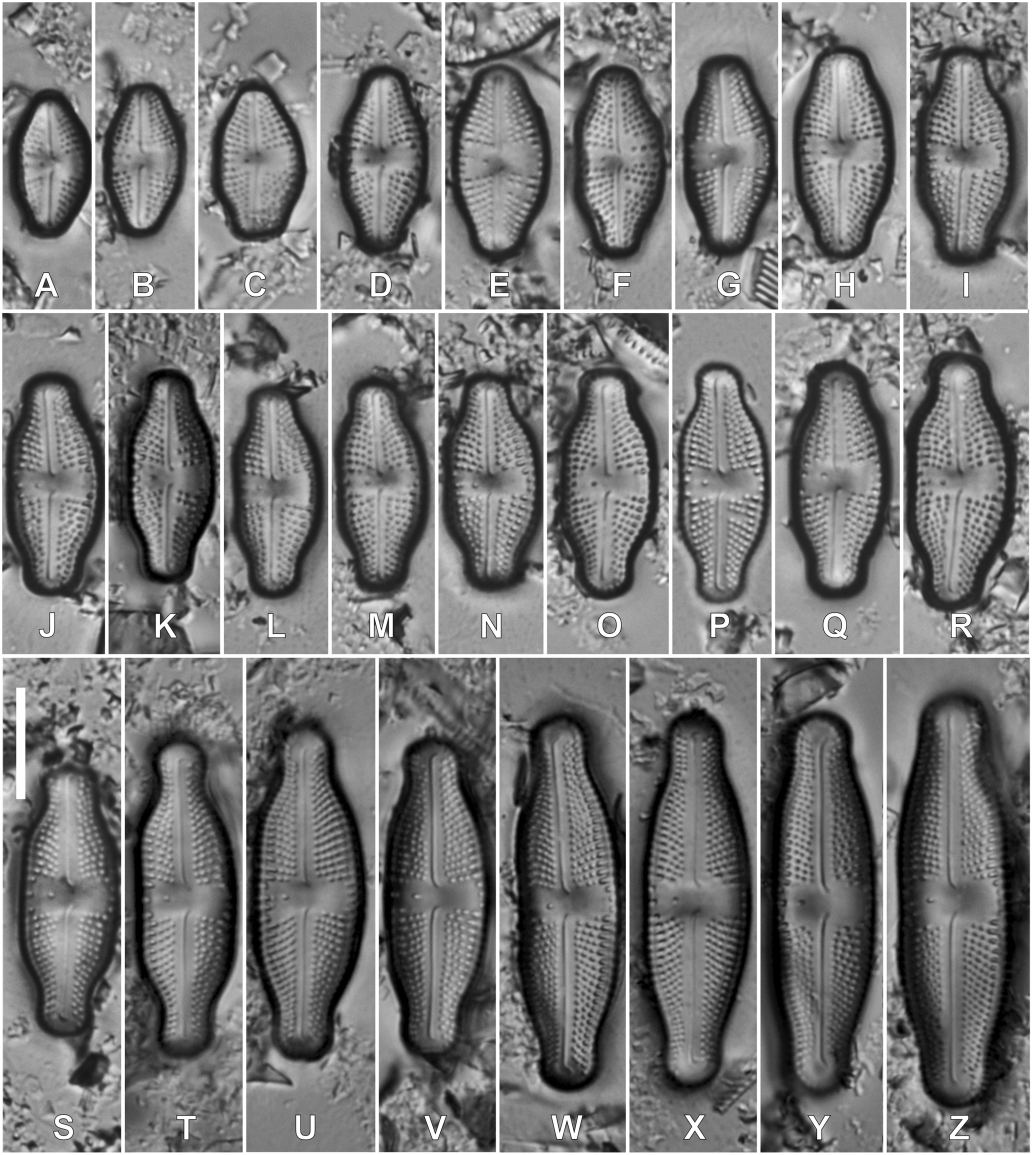
(A–Z) LM pictures of the holotype population of *Luticola kaweckae*. Scale bar represents 10 µm. P Holotype specimen.

**Figure 3:**
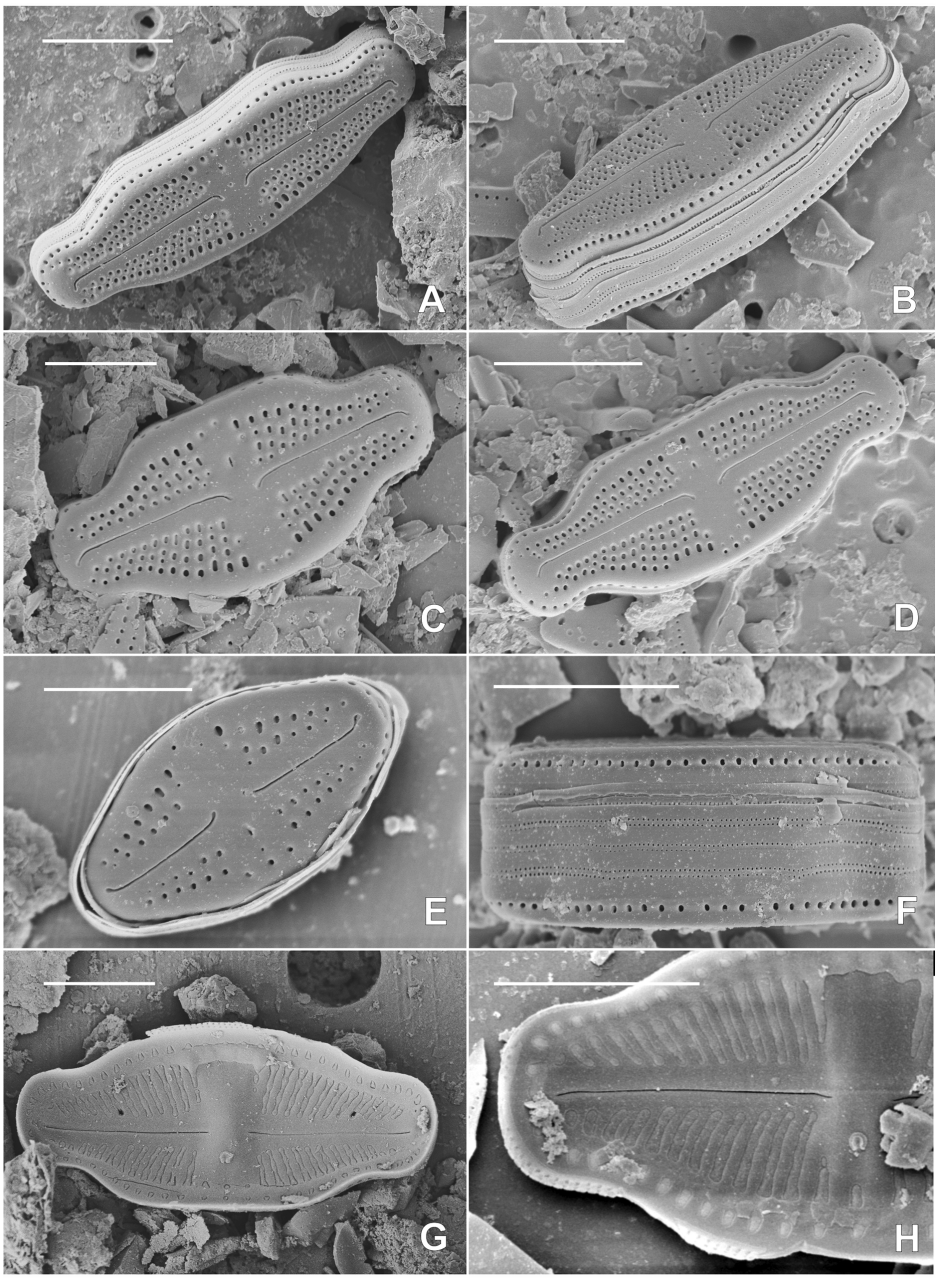
SEM pictures of the holotype population of *Luticola kaweckae*. (A–E) External views of entire valves showing the morphological changes with decreasing valve length; (F) frustule in girdle view; (G) internal view of an entire valve; (H) internal detail of the raphe and the continuous hymenes covering the areolae. Scale bars represent 5 µm.

***Luticola kaweckae* Kochman-Kędziora, Noga, Olech & Van de Vijver, sp. nov**. (Figs. 2, 3).

*Light microscopy* (Figs. 2A–2Z): Larger valves lanceolate to elliptic-lanceolate, showing a clear asymmetry in valve outline along the apical axis. Valve margin (isolated pore-bearing side) straight, often slightly concave (Figs. 2F, 2L and 2W). Opposite valve margin convex. Apices broadly rostrate-capitate to almost capitate in the largest valves. Smaller valves elliptic-lanceolate to rhombic-lanceolate with short, subrostrate apices. Smallest valves (not exceeding 10 µm in length) almost elliptic with very short, varied in shape apices. Initial valve lanceolate with protracted, broadly round apices (Figs. 2Y and 2Z). Valve dimensions (*n* = 190): valve length 8.0–36.0 μm (initial valves up to 45 µm), valve width 5.5–12.0 μm. Axial area linear (larger valves), slightly expanded towards the central area in smaller specimens. Single isolated pore present in the central area, located more or less halfway between the valve margin and center. Central area wide, bordered with 3–4 areolae. In some valves, two rows of areolae bordering the side bearing the isolated pore. Raphe branches straight with deflected central raphe endings and short, deflected, terminal raphe fissures. Striae radiate throughout the entire valve, 14–20 in 10 μm, composed of up to five large areolae, the largest near the valve margins.

*Scanning electron microscopy* (Figs. 3A–3H): Striae uniseriate, composed of three to five areolae (occasionally six in the largest valves), one to two areolae at the apices. Areolae round to transapically elongated, clearly larger close to the valve margins (Fig. 3C). Central raphe endings short, unilaterally deflected to the side opposite the isolated pore. Terminal raphe fissures short, deflected to the same side as the central endings, occasionally with drop-like pores (Fig. 3E). External isolated pore opening rounded to weakly transapically elongated, slitlike (Figs. 3C–3E). Internally, areolae occluded by hymenes, forming a continuous strip over the striae (Figs. 3G and 3H). Central raphe endings straight. Terminal raphe endings terminating on small helictoglossae. Internally, central nodule rounded, isolated pore covered by a c-shaped silica flap (Figs. 3G and 3H). Single row of round areolae, not interrupted at the apices, present on the valve mantle (Figs. 3A and 3B). Two rows of small rounded perforations present on each girdle band (Fig. 3F).

**Figure 4:**
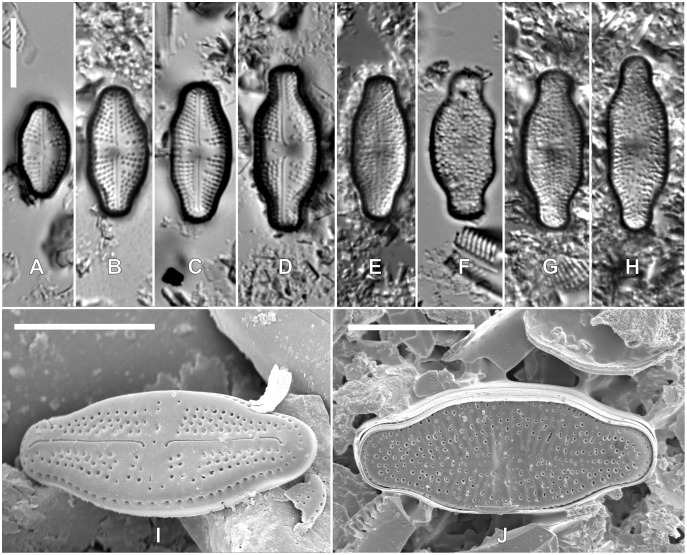
LM (A–H) and SEM (I–J) pictures of teratological forms of *Luticola kaweckae*. Scale bars represent 10 µm.

*Teratological forms:* In samples from nutrient-rich rookeries, most observed specimens showed smaller valve dimensions, although several larger valves could be recorded. Part of the *Luticola kaweckae* valves showed distinct morphological deformities expressed by the lack of raphe, incomplete striae or an irregular areola pattern (Fig. 4). These abnormally shaped valves were not observed in samples lacking this high nutrient input.

**Type locality:** Maritime Antarctic region, South Shetland Islands, King George Island, Windy Glacier forefield, sample Q18.2009 (GPS: 62°14′08.2″S 58°28′15.5″W, *leg*. M. Olech, *coll. date* 2 January 2009).

**Holotype**: holotype designated here, Fig. 2P, slide BR-4719 in Diatom Collection in Meise Botanic Garden, Belgium.

**Isotype**: slide Q18.2009 in the Diatom Collection of the Institute of Agricultural Sciences, Land Management and Environmental Protection at the University of Rzeszów.

**Etymology.** The species is named after Prof. Dr Barbara Kawecka to honor her important contributions to diatom research, including studies in the Antarctic Region, especially on the *Luticola* genus.

**Ecology and distribution:***Luticola kaweckae* is very abundant in soils. The largest populations (up to 97.7% of all counted valves in the sample A1.07) were observed in dry soils clearly influenced by penguins with large guano deposits, partly covered by *Prasiola crispa* (Lightfoot) Kützing (Chlorophyta) on the area of abandoned colony of gentoo penguins (transect A, Ecology Glacier Forefield). Additional large populations of *L. kaweckae* (about 60% of all counted diatoms) were recorded in samples collected from Patelnia Point (Windy Glacier Forefield), also on areas influenced by a chinstrap penguin colony. All major populations were associated with specific soil conditions: an acidic pH and high conductivity. Recently, a population of *L. kaweckae* was observed in the top soil layer, entirely covered by the grass *Deschampsia antarctica*. Occasionally, smaller populations were also present in freshwater habitats (N Kochman-Kędziora, 2009, personal observations).

### Influence of penguin breeding colonies on diatom assemblages

**Figure 5:**
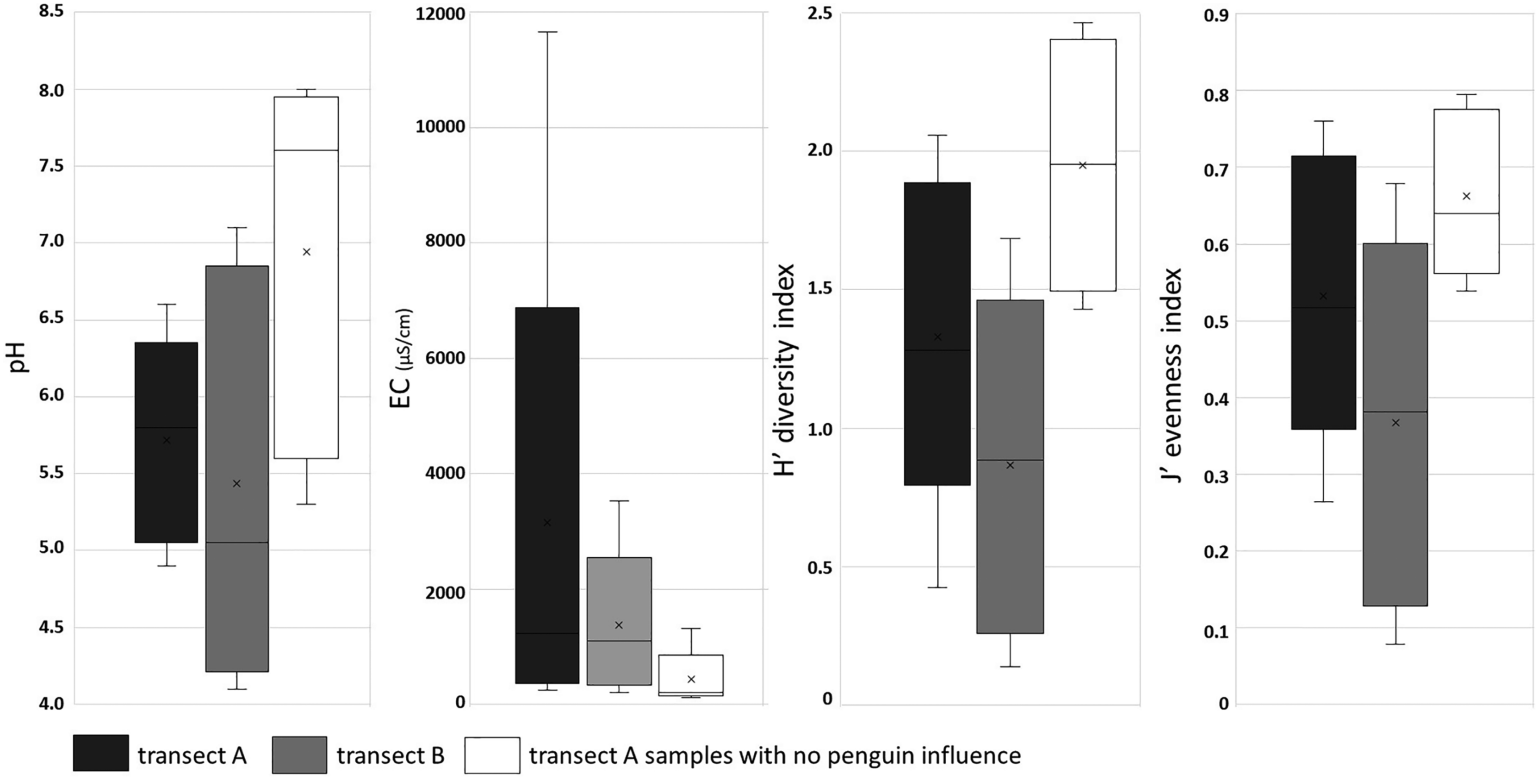
Box plot of soil chemical parameters (pH and EC), and ecological indices: diversity and evenness in three groups of samples. First and third quartiles are drawn using a box, median is shown with a horizontal line and average is shown with x inside the box, minimal and maximal values are shown with whiskers.

The presence of penguin rookeries influenced both environmental parameters and diatom assemblages. Samples collected from all penguin rookeries showed an acid pH, whereas most of the soil samples from more distant sites of transect A had a circumneutral to alkaline pH. The largest differences were observed in the measured conductivity values. Samples from the penguin rookeries (in both transects) were characterized by very high conductivity, exceeding 1,000 µS/cm in all the sites located at the center of the rookeries (Fig. 5). They were also much less diverse in diatom species. The number of diatom taxa in samples collected in or near the penguin rookeries in transect A and B, was only 37 and 35 diatom taxa, respectively. On the other hand, more distant samples of transect A, lacking an elevated nutrient input, almost twice more taxa (71 taxa) were recorded. PERMANOVA showed significant differences between diatom assemblages influenced and non-influenced by penguin rookeries (*pseudo*-F = 4.4933, *P* = 0.001). Differences between these samples could also be noted in the diversity index. The lowest mean diversity was observed for samples collected in the penguin rookery in transect A (0.83 ± 0.55), whereas a slightly higher mean value was observed for transect B (1.35 ± 0.51). On the contrary, samples devoid of direct penguin influence were characterized by a higher mean diversity of 2.0 ± 0.41 (Fig. 5).

In samples from the penguin nesting area (both transects combined), the five most dominant taxa constituted almost 85% of all counted valves with *Luticola kaweckae* (53%) as principal taxon, followed by *Pinnularia borealis* Ehrenberg s.l. (18%), *Luticola puchalskiana* Kochman-Kędziora, Zidarova, Noga, Olech & Van de Vijver (5%), *Chamaepinnularia krookiformis* (4%) and *Pinnularia australoborealis* Van de Vijver & Zidarova (almost 3%). *Luticola kaweckae* was the dominant species, both in number of counted valves and in number of samples present. It formed the largest populations on soils partially covered by algal mats of *Prasiola crispa*, reaching often abundances between 87.5% to even 97.7% (in sample A1.07) of all counted valves. *Pinnularia borealis* formed its largest populations in samples from transect A in 2015. Other species were noted only occasionally and their share in the community was marginal.

Although in both studied transects (A and B), an almost identical number of species was observed, significant differences in diatom assemblages were observed, confirmed by PERMANOVA (*pseudo*-F = 2.4645, *P* = 0.008). Both transects share only 13 non-marine species (Table 1).

Diatom assemblages from transect A (the gentoo penguins *(Pygoscelis papua)* rookery on the Ecology Glacier forefield) were mostly dominated by only two species–*Luticola kaweckae* and *Pinnularia borealis* s.l., whose joint share in the assemblages, especially in 2007, exceeded 90%. In only one sample (A5.07) a more diverse community was observed, composed of *Chamaepinnularia krookiformis* (39%), *Pinnularia microstauroides* (26%), *Mayamaea permitis* (16.6%) and *Pinnularia australomicrostauron* Zidarova, Kopalová & Van de Vijver (8.6%). This sample had the lowest share of *Luticola kaweckae* in the part of transect A influenced by gentoo penguins with only 3.1% of counts.

Contrary, diatom assemblages from transect B (the chinstrap penguin *(Pygoscelis antarcticus)* rookery on the Cape Patelnia) had a different composition. The diatom assemblages in samples with the highest nutrient input (B1–B4) were dominated not by one but several taxa including: *Luticola kaweckae, L. puchalskiana* and various *Pinnularia* species such as *P. austroshetlandica*, *P. australoborealis*, *P. borealis* s.l., *P. australomicrostauron* and *P. australoschoenfelderii* Zidarova, Kopalová & Van de Vijver. Additionally in the sample B1.09, a relatively large population of *Navicula massalskiana* Kochman-Kędziora, Olech & Van de Vijver was observed reaching an abundance of 18.3%. It is noteworthy that *Luticola puchalskiana*, *Pinnularia austroshetlandica* and *Navicula massalskiana* were entirely absent in samples from transect A (both from the penguin rookery and in the non-impacted soil samples).

*Luticola kaweckae* formed large populations, but was less abundant than in transect A. *Pinnularia borealis* s.l. was a dominant species in only two samples (B4.06 and B5.06) collected in 2006 whereas in the other samples (from both 2006 and 2009), the species was much less abundant, or even absent.

Soil samples devoid of direct penguin influence (A6–A15 collected in 2007 and 2015) were characterized by a much higher diatom richness (71 observed taxa in only 20 samples) with half of them (36 taxa) never observed in samples influenced by penguins. With increasing distance from the edges of the penguin colonies, at sites with various vegetation, soil diatom assemblages became more complex, diverse, and the share of *Luticola kaweckae* and *Pinnularia borealis* decreased significantly. The highest diatom diversity with minimum 20 species was noted in samples devoid of direct influence from penguins activity, partially covered mostly by grass *Deschampsia antarctica*. Samples have slightly alkaline pH (7.6–8.0), only in sample A10.15 pH was slightly acid (6.1). *Pinnularia borealis* s.l., recorded in 16 from 20 samples and with abundances up to almost 23% of all counts, was the most dominant species. The largest population was observed in sample A6 in both seasons (2007 and 2015), exceeding 50% of all counted diatoms.

**Figure 6:**
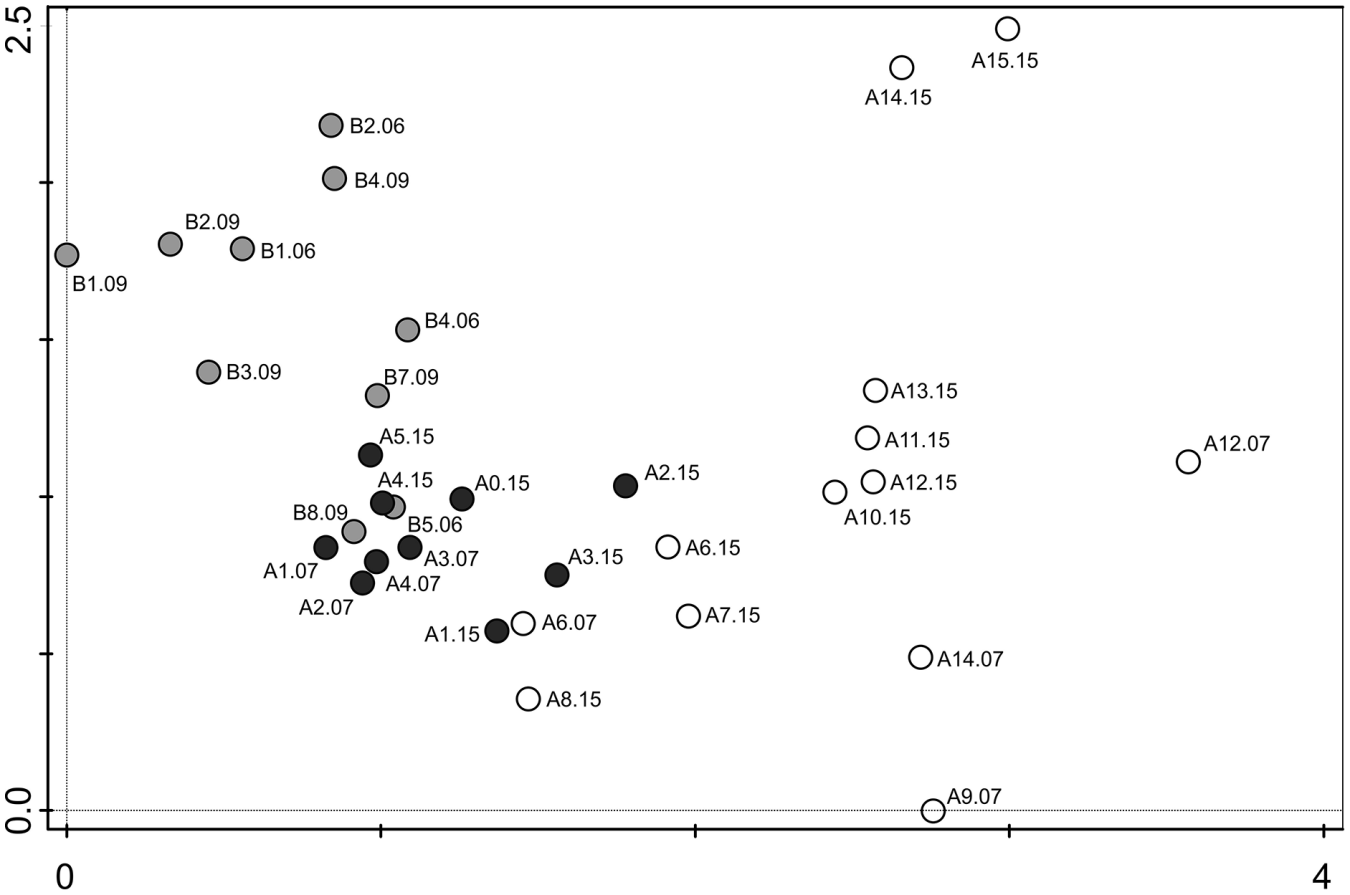
Detrended correspondence analysis (DCA) of the samples from transects A and B. Grey dots–samples collected from transect B (chinstrap penguin rookery), black dots–samples collected from transect A with gentoo penguins influence, white dots–samples from transect A with no penguin influence.

The second most dominant species, *Luticola quadriscrobiculata* Van de Vijver (8.5% of all counted diatoms) was present only in samples devoid of direct penguin influence. It formed large populations in several samples, from both 2007 and 2015 (Fig. 6). It is noteworthy that the dominant species in penguin rookeries *Luticola kaweckae* never formed large populations in samples lacking a high nutrient input. The taxon was only present with more than 10% in a handful of samples.

**Figure 7:**
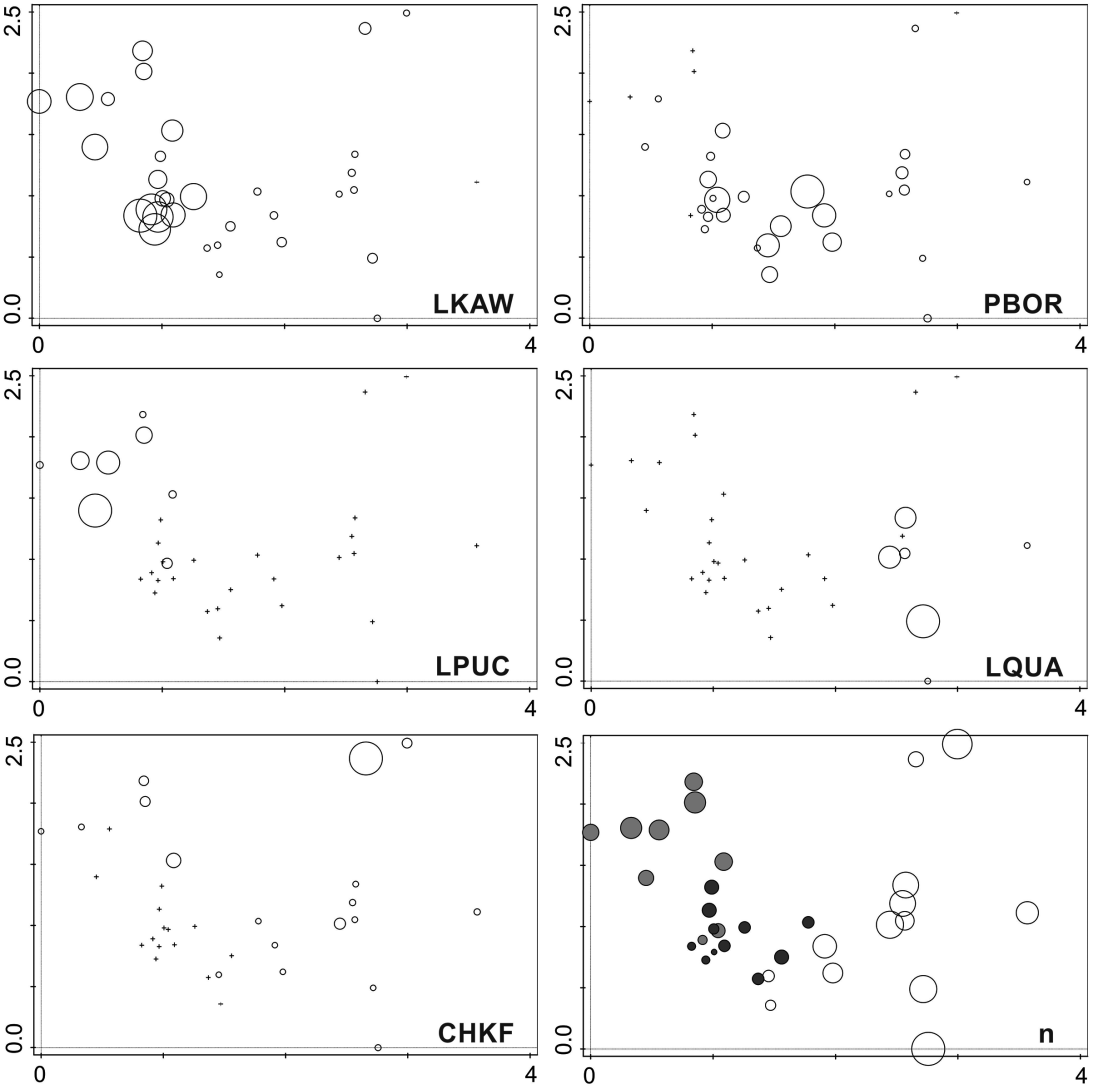
Symbol plots of generated in the DCA ordination space presenting the occurrence of the five dominant species and diversity of investigated samples. LKAW, *Luticola kaweckae*, PBOR, *Pinnularia borealis* s.l., LPUCH, *Luticola puchalskiana*, LQUA, *Luticola quadriscrobiculata*, CHKRF, *Chamaepinnularia krookiformis*, *n*, number of recorded species. The position of the sampling sites is the same as in Fig. 6. The species abundance (for dominant species) and the number of recorded taxa is related to the size of the circles. Cross indicates the absence of the species in the sample. Colors on the plot with the number of species indicate individual research transects (light gray—transect B, dark gray—transect A on the penguin rookery, white—transect A devoid of penguin influence).

The DCA analysis revealed a considerable variability in the diatom data (Fig. 6). The results of the ordination clearly show the influence of the penguin presence on the diatom assemblages, regardless of the sampling season. Samples from transect B (grey symbols) collected from sites situated closest to the penguin rookery, are positioned on the left side. In the center, several samples from transect B taken further away from the penguin rookeries and samples from transect A collected from the penguin rookery or its immediate vicinity (black symbols) are grouped. Thirdly, on the right side of the diagram, all samples collected far from the penguin nesting sites, and thus devoid of direct guano influence (white symbols) are located. Changes in the species composition of diatom assemblages and their similarity in the DCA diagram change continuously along the environmental gradient. Figure 7 shows the changes in the abundance of the five dominant taxa in the symbol plot in DCA ordination space (Fig. 7).

## Discussion

### General diatom richness

In the studied samples a rather high number of identified taxa is expected for soil habitats in the Maritime Antarctic region. In a recent study discussing 13 samples collected from soils in two stream valleys and a dried-up river bed of the Ornithologist Creek, close to the present study area, a total of 98 diatom taxa was recorded (Noga et al., 2020). A lower species richness was observed in soils from the lake-wash zone on James Ross Island. In 21 analyzed wet soil samples 41 diatom taxa were recorded (Kopalova et al., 2019). In 2007, Fermani, Mataloni & Van de Vijver (2007) investigated the soil microalgal communities on the volcanic Deception Island, one of the other major islands of the South Shetland Archipelago. In 18 samples, 140 algal taxa, including 74 diatoms, were recorded. However, since this study was taken before the large taxonomic revision of the diatom flora of the region (Zidarova, Kopalová & Van de Vijver, 2016 and references listed therein), the reported number of taxa most likely does not reflect the actual diatom diversity, and might be underestimated. Only two other studies deal with diatom diversity in soils influenced by animals in the Antarctic region. Mataloni & Tell (2002) analyzed three soil samples collected from penguin colonies at Cierva Point (the Antarctic Peninsula Region). Among other groups of algae, they recorded only six diatom taxa. However, similarly to the study conducted by Fermani, Mataloni & Van de Vijver (2007) it is hardly possible to compare these older studies with the present one, as the reported lower diversity is most likely a consequence of incorrect species identification in the past, based on the similarity to European and North American species, what Tyler (1996) defined as force-fitting. The important taxonomic revision (Zidarova, Kopalová & Van de Vijver, 2016 and the references therein) started in 2010, revealed a higher diatom species richness for the region, also confirmed by the results reported in the present paper.

### Influence of penguins on diatom communities

The acid pH and high conductivity values of samples collected from penguin rookeries are comparable to results published by Zwolicki et al. (2015), where with increasing distance from bird colonies the concentrations of nutrients and conductivity values decreased, whereas pH values increased.

The diversity observed in samples from rookeries and surrounding areas was less than half of that in samples devoid of penguin influence. At sites with the highest nutrient input, covered by *Prasiola crispa* mats, only a few taxa were recorded. This low diatom diversity was also observed in other localities, in all three regions of the Antarctic realm, *i.e*. the Antarctic continent (Ohtani et al., 2000), the sub-Antarctic Islands (Van de Vijver, Ledeganck & Beyens, 2002) and the Maritime Antarctic region (Mataloni & Tell, 2002). It most likely results from the fact that only a small number of species can adapt and benefit from these extreme habitat conditions of enormous nutrient input (Smykla, Wołek & Barcikowski, 2007), as shown by the overall dominance of *Luticola kaweckae*, together with *Pinnularia borealis* s.l., which also explains the low diversity and evenness in these samples. Akiyama, Kanda & Ohyama (1986) investigated the effect of water extracts of soil from penguin rookeries on algal development, demonstrating that the highly concentrated water extract inhibits algal growth and recognizing acrylic and oxalic acids as the main algal growth inhibitors. On the other hand, a diluted extract stimulates the growth of algae. A similar conclusion was reached by Myrcha, Pietr & Tatur (1985), stating that ornithogenic soils formed on decomposed guano, provide one of the few suitable habitats for plants in the most barren areas of Antarctic Region.

A much higher species richness was observed by Moravcová, Beyens & Van de Vijver (2010) in soils influenced by the wandering albatross on the sub-Antarctic Ile de la Possession. In 108 samples a total of 163 diatom taxa were noted. The larger number of species (but also the different diatom communities) observed by Moravcová, Beyens & Van de Vijver (2010), is related to the clear bioregionalism in the diatom flora of the Antarctic realm (Verleyen et al., 2021), and the typical decrease in species richness with increasing latitude (Jones, 1996). Moreover, Smykla, Wołek & Barcikowski (2007) concluded, that the vegetation around individual nests of flying birds is usually not devastated in contrast to huge penguin colonies. In addition to the clear bioregionalism of the sub-Antarctic region, more diverse algal communities in soils around the nests of wandering albatrosses may also result from a lower nutrient input compared to the penguin rookeries. Diatom assemblages become more diverse with increasing distance from the penguin rookeries, as shown by a higher species richness and a higher evenness as several (instead of only one) taxa start to co-dominate. Obtained results confirm an observation made by Mataloni & Tell (2002), who suggested that soil algal biodiversity decreases with increasing trophic status. In turn, in their study on the zonation vegetation pattern in relation to penguin activity, Smykla, Wołek & Barcikowski (2007) demonstrated that both extremely high nutrient enrichment and scarcity of nutrient availability clearly inhibit the development of vegetation, affecting both species richness and diversity. Similar observations were made by Van de Vijver, Ledeganck & Beyens (2002). On a local scale, the development and formation of soil diatom communities depends on a number of different factors, such as humidity, presence of marine birds and mammals, chemical properties of the soil and topographic features (Van de Vijver, Ledeganck & Beyens, 2002).

The latter conclusion is partly confirmed by the results in the present study. The most diverse assemblages were observed in samples collected from the sites furthest away from the penguin rookeries, characterized by moderate to high conductivity values, however, not related to direct penguin influence. Those sites were located in a flat area, or in small, shallow depressions, partially covered by single clumps of *Deschampsia antarctica* and mosses. Vegetation cover stabilizes the soil surface, with additionally mosses absorbing and retaining water, creating relatively stable humidity conditions (Van de Vijver, Ledeganck & Beyens, 2002). Moreover, Kejna, Sobota & Araźny (2012) indicated that strong Antarctic winds may increase evaporation, especially on the tops or windward slopes, intensifying the humidity conditions. The diatom development in soils (as well as on mosses) is strongly dependent on moisture content (Chipev & Temniskova-Topalova, 1999; Van de Vijver & Beyens, 1999; Van de Vijver, Ledeganck & Beyens, 2002). Mosses growing on soils can buffer environmental variables, creating a protected microhabitat suitable for increased algal development (Vinocur & Maidana, 2010), which could partially explain the higher diversity of species in these samples.

Sample A15.15, having one of the highest species numbers, differed from the others in the composition of the assemblage and was dominated by various species from the genera *Achnanthes* Bory and *Chamaepinnularia* Lange-Bertalot & Krammer with a relatively high proportion of *Luticola* species, especially *L. quadriscrobiculata*. The most interesting species *Achnanthes taylorensis*Kellogg et al. (1980), which also dominated sample A15.15, is almost exclusively known from the Antarctic continent (Kellogg et al., 1980; Sabbe et al., 2003). In the Maritime Antarctic region, it was only sporadically reported from seepages and soils on James Ross Island (Kopalová et al., 2012; Chattová et al., 2022), but it maximum abundance did not exceed 2% (Kopalová et al., 2012). Single specimens were also found in the soils of the South Shetland archipelago (Zidarova, Kopalová & Van de Vijver, 2016). However, so far no report from this region has mentioned such a large population as the one observed in the present study (16%), indicating that when suitable conditions prevail, the species can also be more common in the Maritime Antarctic region. *Achnanthes taylorensis* seems to prefer soils with high conductivity associated with sea salt (Chattová et al., 2022), hence its high abundance in the sample A15.15 located close to the seashore.

Sample A5.07, removed from the DCA analysis as outlier, was co-dominated by three species: *Pinnularia microstauroides*, *Mayamaea permitis* and *Chamaepinnularia krookiformis*, reaching 26%, 16.6% and 36.9%, respectively. The first two species did not dominate in any other sample, and are primarily observed in aquatic or moss habitats (Zidarova, Kopalová & Van de Vijver, 2012, 2016). A5.07 was located on the slope of a moraine, had a high conductivity (1702 µS/cm). It is highly likely that a small, shallow pond was present on this locality, which dried up in a short time. This would explain the contribution of both aerophilic and typically aquatic diatom taxa. Similarly, at site A12.07, located in a depression, *Navicula gregaria* Donkin dominated (60.9%) followed by *Navicula australoshetlandica* Van de Vijver. *Navicula gregaria* is a cosmopolitan species (Lange-Bertalot, 2001), widespread also in Antarctica, where it is found in various, almost exclusively aquatic habitats (Van de Vijver et al., 2011). It can develop in soils, but then only in very small numbers (Zidarova, Kopalová & Van de Vijver, 2016). Since such large populations as observed in sample A12.07 are typical for aquatic habitats, it is also likely that a small pool was present on this spot one day. *Navicula gregaria* was found in a few other samples, but never formed large populations.

Although the samples from two different penguin colonies were dominated by the same species, important differences in species composition could be observed. Three species, *Luticola puchalskiana*, *Pinnularia austroshetlandica* and *Navicula massalskiana*, formed relatively abundant populations in samples influenced by the chinstrap penguin rookery (transect B), but were not observed in samples from the gentoo penguin rookery and samples lacking nutrient input. Although it is unclear what causes this difference, we could speculate that the close proximity to the sea, but also penguin diet may play an important role. Zwolicki et al. (2015) noted that differences in food preferences of various penguin species potentially have a different impact of soil chemistry. Both studied transects differed in basic soil properties (especially EC), but it is impossible to prove in this study whether the differentiation in soil properties is the reason for the differences in the occurrence of some diatom species.

### The occurrence of *Luticola kaweckae* and its resemblance to other Antarctic species

*Luticola kaweckae*, the most abundant species in the investigated samples, described herein as a new species, was not observed in other Antarctic localities. Nevertheless, its distinctive ecological preferences suggest it is probably a more widespread species, than currently known. Most likely, the valves from samples with penguin influence identified as *L. muticopsis* by Mataloni & Tell (2002) may, in fact, represent *L. kaweckae*. A reanalysis of these samples together with a review of other samples from similar habitats will be necessary to confirm the more widespread distribution of this new species.

For many years, *Luticola muticopsis* was a catch-all species complex for capitate *Luticola* species. However, following the revision of the non-marine Antarctic diatom flora (Zidarova, Kopalová & Van de Vijver, 2016 and the references therein), a large number of capitate *Luticola* species have been separated from *L. muticopsis* s.l. and therefore, these species need to be compared with *L. kaweckae* to justify its separation as independent taxon. The new species shows a high degree of similarity with several capitate *Luticola* species due to overlap in valve dimensions and stria density: *L. muticopsis*, *L. austroatlantica* Van de Vijver et al., *L. permuticopsis*, and *L. truncata*. Table 2 presents a comparison of these taxa with *L. kaweckae*.

**Table 2:** Table of comparisons of most similar capitate *Luticola* taxa from the Antarctic region.

	*L. kaweckae* sp. nov.	*L. muticopsis*	*L. muticopsis* (Antarctic Continent)	*L. austroatlantica*	*L. permuticopsis*	*L. truncata*
**Length (µm)**	8.0–36 (45)	19.0–29.0	13.9–31.4	13.5–30.0	18.5–29.0	16.0–40.0
**Width (µm)**	5.4–12.0	8.5–10.5	6.7–10.2	6.0–8.0	7.3–10.5	7.0–10.5
**Striae (in 10 µm)**	14–20	15–16	15–19	15–18, in smallest valves up to 20	18–22	15–19
**Symmetry**	Asymmetrical	Asymmetrical	Asymmetrical	Symmetrical	Symmetrical	Symmetrical
**Valve outline**	Valves lanceolate to elliptic-lanceolate, becoming more rhombic-lanceolate in smaller specimens	Rectangular to broadly elliptic	Linear-elliptical	Linear-lanceolate, smaller specimens elliptic-lanceolate	Linear-lanceolate to linear-elliptic	Valves lanceolate to elliptic-lanceolate smaller elliptic-lanceolate to elliptic
**Margins**	pore-bearing side straight, slightly concave in the largest specimens, opposite margin convex	Isolated pore-bearing side straight, opposite valve margin convex	pore-bearing valve margin straight, the opposite convex	Both margins clearly convex, occasionally the margin of the isolated pore side less convex than the other	Both margins distinctly convex	Weakly undulating, convex margins, in smaller valves the margin of the pore-side less convex
**Apices**	Broadly rostrate-capitate, in the largest valves almost capitate, in the smaller valves short subrostrate	Rostrate to rostrate-capitate, in some populations distinctly capitate	Broadly capitate, becoming truncate in smaller specimens	protracted capitate, in smaller valves rostrate or subrostrate	Broadly rounded, clearly capitate	In larger valves subcapitate to rostrate-subcapitate, in smaller valves truncated
**Raphe** **structure**	Raphe branches straight, both small, drop-like endings simply bent	Raphe straight with indistinct central and distal raphe endings, both weakly bent	Raphe straight, both raphe endings strongly deflected	Central raphe endings weakly deflected, drop-like expanded; distal raphe fissures short, slightly deflected, drop-like expanded	Central raphe endings slightly expanded; distal raphe fissures clearly elongated, deflected	Central raphe endings weakly deflected; terminal fissures very short
**Areolae** **per striae**	Usually 3–5(6), in smaller valves and near the apices 1–2	2–6 almost rounded areolae	3–5, at apices 1–3, areolae rounded, becoming elongated at valve margins	2–3(4) areolae near the axial area rounded, near the margin transapically elongated	3–6 moderately large, rounded areolae, enlarged near the margin.	2–5 rounded to elongated
**Areolae** **on the mantle**	One row, not interrupted at the apices	One row, not interrupted at the apices	One row, not interrupted at the apices,	One row, interrupted at the apices	One row, interrupted at the apices	One row, interrupted at the apices

Among them, *Luticola kaweckae* shows the highest degree of similarity to *Luticola truncata*, distinguished from the *Luticola muticopsis* group in 2009 (Kopalová et al., 2009), but can be separated from all other species by a combination of valve outline and symmetry, shape of the margins, apices, raphe structure and areola pattern on the mantle (Table 2). Valves of *L. kaweckae* are typically asymmetrical, lanceolate to elliptic-lanceolate, becoming more rhombic- lanceolate in smaller specimens. Three species, *L. austroatlantica*, *L. permuticopsis* and *L. truncata*, show very symmetrical valves, with linear-lanceolate valve outlines (becoming more elliptic in smaller specimens). Moreover, the valve margins of both *L. austroatlantica* and *L. permuticopsis* are distinctly convex, contrary to the almost straight, asymmetrical margins of *L. kaweckae* (Kopalová et al., 2011; Esposito et al., 2008; Zidarova, Kopalová & Van de Vijver, 2016). Both species also possess characteristic, distinctive apices: capitate and broadly rounded in *L. permuticopsis* and smaller, protracted, capitate (subrostrate in smaller valves) in *L. austroatlantica*. Apices in *L. kaweckae* are broadly rostrate-capitate in larger valves, becoming short, subrostrate in smaller specimens. Finally, the single row of areolae on the valve mantle (visible in SEM), is not interrupted at the apices in *L. kaweckae*, whereas these areolae on the mantle in *L. permuticopsis*, *L. austroatlantica*, but also in *L. truncata* show a small interruption (Kopalová et al., 2009, 2011; Esposito et al., 2008; Zidarova, Kopalová & Van de Vijver, 2016).

Another morphological feature distinguishing *L. kaweckae* from *L. truncata* is its valve outline. The isolated pore-bearing side of *L. kaweckae* is straight (in larger specimens visibly concave) whereas the opposite margin is convex. Both valve margins of *L. truncata* are convex with larger valves margins symmetrically undulating. Additionally, the shape of the apices in *L. kaweckae* varies depending on the valve size. The largest valves have almost capitate apices, becoming broadly rostrate-capitate in the medium-sized specimens and short subrostrate in the smallest. *L. truncata* has subcapitate to rostrate-subcapitate apices, truncated in smaller valves. Both species differs also in their raphe structure: *L. truncata* has very short terminal raphe fissures, and weakly deflected central raphe endings. Contrary, both raphe endings of *L. truncata* are bent with drop-like pores (Kopalová et al., 2009, Zidarova, Kopalová & Van de Vijver, 2016). Three distinct features separate *Luticola kaweckae* and *Luticola muticopsis*: shape of apices and valves margin, as well as valve outline (Table 2). The most important seems to be the shape of the apices. All specimens of *L. muticopsis*, regardless of their size, have rostrate-capitate to distinctly capitate apices (Van de Vijver & Mataloni, 2008; Kohler et al., 2015), whereas the shape of apices in *L. kaweckae* is never clearly capitate, but almost always broadly rostrate-capitate in larger valves, and very short, subrostrate, often irregular in the smaller specimens. An additional morphological feature characteristic for *L. kaweckae* is the shape of the valve margin on the isolated pore-bearing side. Especially (but not only) in larger valves, the valve margin is slightly concave, whereas the pore-bearing valve margin of *L. muticopsis* is always straight, never concave or convex. Finally, both species differ in their valve outline: rectangular to broadly elliptic in *L. muticopsis* and from lanceolate, elliptic-lanceolate to rhombic-lanceolate in *L. kaweckae* (Van de Vijver & Mataloni, 2008; Kohler et al., 2015). In Van de Vijver & Mataloni (2008, figs 100–103), a taxon, most likely conspecific with *L. kaweckae*, was identified as *Luticola* aff. *muticopsis* 2, a taxon only found in a chinstrap penguin rookery.

The range of observed dimensions was unusually wide in the new species. In the studied soil samples from the penguin rookeries, valves of the entire size spectrum were noted, from large initial cells with a length exceeding 30 µm, as well as small ones with a length less than 10 µm. Diatoms developing in the surface soil layer in the Antarctic region are exposed to extremely harsh and unfavorable conditions: water deficiency, low temperatures, extremely low or high nutrient content. The species composition as well as the shape and size of diatom cells are strongly influenced by the habitat conditions, especially moisture content (Van de Vijver, Ledeganck & Beyens, 2002; Spaulding et al., 2010). Moreover, only a small number of diatom species is able to survive in these xeric conditions (Van de Vijver & Beyens, 1999), and their cell size decreases with decreasing humidity. For example, *Pinnularia borealis* Ehrenberg valves observed on South Georgia were significantly smaller in samples collected from dry mosses than from sites where the mosses were wetter (Van de Vijver & Beyens, 1997; Spaulding et al., 2010).

It is possible that the observed cell size reduction in *L. kaweckae* in samples with the largest penguin influence, is a specific protective response mechanism against environmental stress conditions such as moisture limitation and unusually elevated nutrient input. This extremely high nutrient input near the penguin rookery could be one of the presumable causes of the occurrence of abnormal morphological forms of *L. kaweckae* with visible malformations in the structure of the valves. Generally, teratological forms are an accidental effect of environmental stress, such as the influence of heavy metals, physical stress or pesticides, which emerge as deviations from normal shape or ornamentation (Lavoie et al., 2017; Falasco et al., 2021 and references therein). In Antarctic diatoms morphological deformities are observed rarely, usually in single specimens (N. Kochman-Kędziora, 2014–2020, personal observations). Only one paper from the Antarctic Region reported teratological modifications in *Pinnularia obaesa* Van de Vijver striae structure, which may result from periods of drought (Van de Vijver, 2008).

## Conclusions

Our study showed that areas influenced by penguins are more diverse, although samples from nutrient-rich rookeries are dominated by only a limited number of species with often an overall domination of the newly described species, *Luticola kaweckae*.

It is also clear from the results of the present study, that the disturbance associated with the presence of penguin rookeries, clearly alters the soil diatom diversity in this area masking the original, non-disturbed composition. Together with previous studies from this area (Noga et al., 2020) it can be concluded that the number of soil diatom species in this area is relatively high. However, the composition of the diatom assemblages on local scale, depends on both biotic (*e.g*. penguin nutrient input) and abiotic (*e.g*. topographic features) conditions.

This article discussed in more detail the diatom assemblages influenced by penguins activity on the breeding places on King George Island. Unfortunately, due to the observed differences between the two penguin colonies, it is too early to draw general conclusions about diatom communities typical of penguin colonies. More research will be needed to get a more precise insight into the interactions within this unique habitat. Based on the obtained results, it is clear that *Luticola kaweckae* is one of the characteristic species for terrestrial habitats with strong penguin influence.

## Supplemental Information

10.7717/peerj.13624/supp-1 Supplemental Information 1 Raw data of measurements made for 130 specimens of the newly species described. The length and width of the valves and the number of striae in 10 µm.

10.7717/peerj.13624/supp-2 Supplemental Information 2 Values of measured parameters: EC and pH.

## Additional Information and Declarations

### Field Study Permissions

The following information was supplied relating to field study approvals (*i.e*., approving body and any reference numbers): An official permit to enter the ASPA 128 (permission no. 05/2014) for Antarctic Expedition 2014/2015 was granted by the Instytut Biochemii I Biofizyki, Polskiej Akademii Nauk, ZAKŁAD Biologii Antarktyki (Institute of Biochemistry and Biophysics, Polish Academy of Sciences, Department of Antarctic Biology).

### Data Availability

The following information was supplied regarding data availability: The raw data is available in the Supplemental Files.

### New Species Registration

The following information was supplied regarding the registration of a newly described species: *Luticola kaweckae*.
